# Sorption, persistence, and leaching of the allelochemical umbelliferone in soils treated with nanoengineered sorbents

**DOI:** 10.1038/s41598-019-46031-z

**Published:** 2019-07-05

**Authors:** Miguel Real, Beatriz Gámiz, Rocío López-Cabeza, Rafael Celis

**Affiliations:** Instituto de Recursos Naturales y Agrobiología de Sevilla (IRNAS), CSIC, Avenida Reina Mercedes 10, 41012 Sevilla, Spain

**Keywords:** Nanoscience and technology, Environmental chemistry

## Abstract

Coumarins represent an important family of allelochemicals with fungicidal, bactericidal, insecticidal, nematicidal, and herbicidal properties. Like for other allelochemicals, the short persistence of coumarins in soils can reduce their biological activity and hamper their application as environmentally friendly agrochemicals. We evaluated the sorption of the coumarin umbelliferone by eight soils and six sorbent materials, and then selected two nanoengineered sorbents, hexadecyltrimethylammonium-modified Arizona montmorillonite (SA-HDTMA) and olive-mill waste biochar (BC), to assess the effect of their addition to two distinct soils on umbelliferone sorption, persistence, and leaching. Umbelliferone was sorbed to a greater extent by the acid soils (A1-A2, *K*_d_ > 4.0 L kg^−1^) than by the alkaline soils (B1-B6, *K*_d_ < 0.5 L kg^−1^). The addition of BC and SA-HDTMA at a rate of 4% to alkaline soil (B2) increased the umbelliferone sorption *K*_d_ value from 0.3 to 1.6–2.0 L kg^−1^, whereas their addition to acid soil (A1) increased the *K*_d_ value from 4.6 to 12.2–19.0 L kg^−1^. Incubation experiments showed that BC had more impact than SA-HDTMA on the persistence of umbelliferone in the soils, increasing its half-life from 0.3-2.5 to 1.2–14.4 days, depending on the soil. Furthermore, the addition of BC to the top 0–5 cm of soil columns reduced leaching of umbelliferone and led to accumulation of umbelliferone residues in the top 0–5 cm soil layer. The addition of nanoengineered materials, such as organoclays and biochars, could thus be a suitable strategy to increase the persistence and reduce the mobility of coumarins in the rhizosphere with the aim of prolonging their biological activity.

## Introduction

The continued increase in the global demand for food relies on the use of synthetic pesticides to achieve high yields of crop production. However, there is also growing public concern about the use of synthetic pesticides in agriculture, because of the risks these substances can pose to the environment and human health^1,2^. For this reason, safer and environmentally friendly alternative crop protection strategies are gaining attention to reduce the current dependence on synthetic pesticides.

Allelopathy has received increasing attention in recent years in the field of crop protection^3–5^. Allelopathy is an adaptive strategy whereby plants (or other organisms) release biochemicals, known as allelochemicals, that influence the growth, survival, development, and reproduction of other organisms^3^. Allelochemicals can be used as natural pesticides to overcome problems such as resistance development and contamination of environmental compartments caused by the indiscriminate use of synthetic pesticides^3,6,7^.

**Figure 1:**
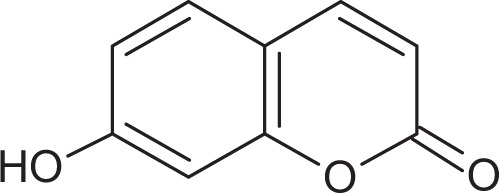
Chemical structure of umbelliferone.

Coumarins represent an important group of allelochemicals. They are widespread in nature and have a broad range of biological activities, e.g., phytotoxic, antifungal, insecticidal, antibacterial, and nematicidal activity^8–14^. Umbelliferone or 7-hydroxycoumarin (Fig. 1) is a naturally occurring coumarin widely distributed in the *Rutaceae*, *Apiaceae* (*Umbelliferae*) and *Asteraceae* families^15–17^. Inhibitory effects of umbelliferone to plants such as *Festuca rubra*, *Medicago sativa* and *Lactuca sativa* have been reported^16,18^. Antifungal and antibacterial properties for umbelliferone and its derivatives have also been described^13,17^. Due to these inhibitory activities, umbelliferone can potentially be considered a natural alternative to certain synthetic pesticides.

The use of allelochemicals as natural pesticides may, however, have some drawbacks. Factors such as their mobility in soils can affect their bioavailability to the target organism and hence their efficacy. For example, Xiao *et al*.^19^ showed that vertical leaching impacted the effect of coumarins on the germination rate of lettuce. Furthermore, allelochemicals usually have short environmental half-lives, because they possess chemical structures that are easily biodegradable in the environment^20,21^. Soil degradation experiments conducted by Guo *et al*.^22^ showed complete biodegradation of umbelliferone after incubation for 2 days. Nanotechnology can help circumvent some of these limitations associated with the use of natural products as agrochemicals by allowing the development of new tools to improve their effectiveness^21,23,24^. For example, some studies have shown that soil amendments, including certain nanoengineered additives, can greatly influence pesticide mobility and bioavailability, and consequently, the final pesticide efficacy^25–28^. Biochars and organoclays can be included among such additives.

Biochar (BC), a product of the pyrolysis of biomass, has been investigated as soil amendment because of its potential to increase the fertility of soils by improving soil properties such as pH, cation exchange capacity, and water-holding-capacity^29–31^. In particular, converting waste biomass into biochar constitutes an effective solution for the safe and beneficial disposal of a number of materials^31^. By adjusting the conditions of the production process, nanoengineered BCs with different surface and porosity nanoscale properties can be obtained, allowing the design of BC samples to match specific applications, including sorption-related applications^26,30,32^. For example, BCs prepared at high pyrolysis temperatures (>500 °C) consist of more graphitised (aromatic) material, with higher specific surface area (SSA) and reduced abundance of surface functional groups, whereas those prepared at lower temperatures have lower SSA and more surface functionality^28^. The molecular structure evolution and micropore development with heat treatment temperature will determine the performance of low- and high-temperature BCs as sorbents of organic compounds and the binding mechanisms involved in the sorption process^33^.

Organoclays are nanoengineered phyllosilicates in which organic modifiers (e.g., alkylammonium cations) are used to change the nature of the clay mineral surface from hydrophilic to hydrophobic, usually to improve its affinity for organic compounds^34^. They have been suggested as amendments to modulate the behaviour of pesticides in soils^25^ and proven, in many studies, to be effective sorbents for different types of organic chemicals, mainly by allowing hydrophobic-type interactions between the sorbate and the organic modifier^35,36^. By selecting the nature of the clay mineral and the size and functional properties of the organic modifier, organoclays with selective affinities for targeted organic compounds can be obtained^37–39^. Organic modifiers can even be directly added to clay-rich soils to improve their sorptive properties for organic compounds by the *in situ* formation of organoclay complexes^40^.

The objective of this study was to assess the potential of different sorbents, including two nanoengineered sorbents, as soil amendments to reduce potentially rapid degradation and transport losses of the coumarin umbelliferone in soil, as a way to prolong its bioactivity. Three phyllosilicates (kaolinite, illite, and montmorillonite), an organo-montmorillonite (SA-HDTMA), and an organic waste (OW) and its biochar (BC) were tested as sorbent materials. The underlying hypothesis was that organic modification of clay minerals and pyrolysis of biomass (olive-mill waste) could be good strategies to increase the sorption of umbelliferone by these raw materials mainly by favouring hydrophobic interactions between the chemical and the sorbents, as previously reported for different synthetic pesticides^36,38,41,42^. To achieve the aim of this work we conducted: (i) preliminary stability experiments to determine suitable conditions for umbelliferone storage and analysis, (ii) sorption tests with eight soils and the six sorbent (amendment) materials to select soils and amendments for further experiments, and (iii) sorption, dissipation, and leaching experiments with two soils and the nanoengineered sorbents (SA-HDTMA and BC) to assess the effect of these on umbelliferone sorption, persistence, and mobility in the soils. The information reported in this paper should be useful to implement the use of coumarins as natural pesticides and take advantage of their allelopathic properties with the help of nanotechnology.

## Materials and Methods

### Chemical

The standard and diluted working solutions of umbelliferone used in this work were obtained from a 200 mg L^−1^ umbelliferone stock solution prepared in methanol. Pure analytical umbelliferone (purity > 99%) purchased from Sigma-Aldrich was used to prepare the stock solution. Umbelliferone has a molecular mass of 162.1 and reported p*K*_a_ and aqueous solubility values of about 7.5 and 620 mg L^−1^, respectively^43,44^.

### Soils

Samples of two acid (A1-A2) and six alkaline (B1-B6) soils from southwestern Spain were used in this study. All soil samples were collected from a 0–20 cm depth, air-dried, sieved to pass a 2 mm mesh, and stored at 4 °C prior to use. The main characteristics of the soils are summarised in Supplementary Table S1.

### Sorbents

The six materials assayed as sorbents of umbelliferone were three phyllosilicates (kaolinite, illite, and montmorillonite), an organo-montmorillonite (SA-HDTMA), and an organic waste (OW) and its biochar (BC). The phyllosilicates, used as supplied by The Clay Minerals Repository of The Clay Minerals Society (Purdue University, USA), were KGa-2 kaolinite (Georgia, USA), IMt-1 illite (Montana, USA), and SAz-1 montmorillonite (Arizona, USA). Detailed physicochemical data of these clays can be found in the Clay Minerals Society webpage^45^. The organo-montmorillonite (SA-HDTMA) was prepared by treating a portion of SAz-1 montmorillonite (4 g) with an aqueous solution (200 mL) containing an amount of hexadecyltrimethylammonium (HDTMA) cation (chloride salt) corresponding to 100% of the cation exchange capacity of SAz-1 (1200 mmol_c_ kg^−1^). It is known that modification of high-charge montmorillonites, such as SAz-1, with HDTMA cations provides these clay minerals with a wide hydrophobic interlayer phase and positively charged ammonium groups, which increase their affinity for organic compounds, including anionic and acid compounds^46,47^. The organic waste (OW) and its biochar (BC) were, respectively, a composted olive-mill waste supplied by an olive-processing factory located in Jaén (Spain) and its pyrolysis product, which was obtained by heating a sample of OW at 400 °C for 4 h under a flow of N_2_ at 1.5 L/min^41^. Olive-mill waste, a by-product of the extraction of olive oil, is generated in high quantities in olive oil-producing countries, and has been proposed as a pesticide biosorbent in its raw, composted, and pyrolysed forms^26,41,42^. Prior to use, the OW was air-dried and ground to pass a 2 mm-aperture sieve. For the preparation of BC, we selected a low pyrolysis temperature to increase the fraction of condensed organic matter while maintaining surface functionalities, because these surface characteristics are known to increase the bioavailability of sorbed compounds compared to biochars prepared at high pyrolysis temperatures^28^.

### Detailed characterisation of SA-HDTMA and BC

The X-ray diffractogram of SA-HDTMA was recorded using a D-5000 diffractometer (Siemens, Germany) with CuK_α_ radiation. The scanning electron micrograph of BC was obtained using a dual beam Zeis Auriga Scanning Electron Microscope (Microscopy Service, University of Seville). Carbon content measurements were conducted using a Leco Truspec CHNS Micro Analyser (Microanalysis Service, University of Seville). Fourier-transform infrared (FT-IR) spectra were recorded using a Tensor II FT/IR spectrometer (Microanalysis Service, University of Seville) on samples diluted (1%) in KBr disks.

### Umbelliferone stability experiments

The impact of light exposure and presence of soluble soil components on the storage stability of umbelliferone was studied for umbelliferone aqueous solutions containing different amounts of methanol, which was used as a stabiliser. High concentrations of alcohol have antimicrobial properties^48^ and previous experiments had shown that the addition of methanol helped stabilise aqueous solutions of readily biodegradable organic compounds, presumably by eliminating microbial activity^24,49^. For the stability experiments, 2 mg L^−1^ umbelliferone solutions (100 mL) were prepared either in distilled water or in a soil extract. The soil extract was obtained by shaking 50 g of B2 soil (the soil in which umbelliferone displayed lower persistence) with distilled water (100 mL) for 24 h, centrifuging, and filtering the supernatant solution with filter paper (Whatman No. 1). Each umbelliferone solution contained different amounts of methanol (1, 10, and 50%) and was either exposed to or protected from light for a period of 8 days. At t = 0, 1, 4, 6 and 8 days the solutions were sampled, filtered, and immediately analysed by high performance liquid chromatography (HPLC) to determine the amount of umbelliferone remaining in solution.

### Sorption experiments

Sorption of umbelliferone on unamended and amended soil samples and on the sorbent materials was determined by batch sorption experiments using glass centrifuge tubes lined with Teflon caps. In triplicate, 8 mL of a 2 mg L^−1^ umbelliferone aqueous solution (C_ini_) was added to samples of the unamended soils (1 g), sorbent materials (20 mg), or soils amended with selected sorbents (BC and SA-HDTMA) at a rate of 4% (40 mg g^−1^). Assuming a soil bulk density of 1.2 g cm^−3^, this rate of BC and SA-HDTMA would be achieved by a field dose of 5 t ha^−1^ for an incorporation depth of 1 cm, or 50 t ha^−1^ for an incorporation depth of 10 cm. This range (5–50 t ha^−1^) coincides with that for which organic amendments such as biochars have been reported to have positive effects on crop yields, with appropriate nutrient management^50^. To avoid the degradation of umbelliferone during the sorption experiment, the tubes containing unamended or amended soil samples were pre-sterilised by autoclaving for 20 min at 121 °C and 200 kPa three times on consecutive days, with 24 h incubation periods at 25 °C between successive autoclaving cycles. After adding the umbelliferone solution, which was also subjected to one autoclaving step, the suspensions were shaken in the dark for 24 h, centrifuged, and then 6 mL of supernatant solution was removed, stabilised with 6 mL of methanol, and then filtered and analysed by HPLC in order to determine the solution concentration of umbelliferone in the equilibrated suspensions, C_e_ (mg L^−1^). The amount of umbelliferone sorbed, C_s_ (mg kg^−1^), was calculated from the difference between the initial (C_ini_) and the equilibrated (C_e_) solution concentration as C_s_ = (C_ini_ − C_e_) × V/m, where V (L) is the volume of solution and m (kg) is the mass of solid. Initial umbelliferone solutions without solids were also shaken for 24 h and indicated no losses of organic compound by sorption to the tubes or to the filter material. Distribution coefficients, *K*_d_ (L kg^−1^), were calculated as *K*_d_ = C_s_/C_e_, whereas sorption percentages were calculated as %Ads = [(C_ini_ − C_e_)/C_ini_] × 100.

To confirm the indirect sorption measurements, these were compared with sorbed amounts directly determined by extraction. For this purpose, the 6 mL of supernatant solution removed for the indirect sorption analysis was replaced with 6 mL of methanol to promote desorption of the umbelliferone previously sorbed by the soils or sorbents. The suspensions were shaken for 24 h, centrifuged, filtered, and the concentration of umbelliferone in the methanolic extract (C_ext_) was determined by HPLC. The amount of umbelliferone desorbed from the solids upon extraction (mg kg^−1^) was then calculated as C_s-dir_ = (C_ext_ − C_e_/4) × V/m.

Samples of SA-HDTMA and BC (10 mg) were also treated with 8 mL of a concentrated umbelliferone aqueous solution (ca. 200 mg L^−1^) and the sorption products were analysed by FT-IR to get insight into the sorption mechanisms. The suspensions were shaken for 24 h, the supernatant was removed and analysed by HPLC, and the solids (sorbent-umbelliferone complexes) were dried at 60 °C and analysed by FT-IR spectroscopy as samples diluted (1%) in KBr disks.

### Dissipation experiments

The effect of the addition of SA-HDTMA and BC on the dissipation of umbelliferone in selected soils (A1 and B2) was studied by an incubation experiment conducted in 10 mL-glass centrifuge tubes. Samples of 1 g of the non-sterile soils, either unamended or amended with SA-HDTMA or BC at a rate of 40 mg g^−1^ soil, were added to the tubes and then treated with an aqueous solution of umbelliferone with appropriate concentration to give an application rate of 2 mg umbelliferone kg^−1^ soil and a water content of 35% (soil A1) or 30% (soil B2). These water contents were selected for being close to the water holding capacity of each soil. Next, the tubes were closed and incubated in the dark at 25 °C. Separate triplicate tubes were incubated for each sampling time (t = 0 to 11 days), to be destructively removed from the incubator and immediately frozen until analysed. Duplicate sterile treatments were also included to control for abiotic umbelliferone breakdown; these consisted of autoclaved unamended soil samples (20 min at 121 °C and 200 kPa, three times on consecutive days) spiked with a sterilised umbelliferone solution as described above for the non-sterile treatments.

For each sampling time, umbelliferone soil residues were determined by extraction with 5 mL of an 80:20 methanol:aqueous H_3_PO_4_ (pH = 2.2) mixture by shaking for 24 h. The suspensions were centrifuged, filtered, and the extracts analysed by HPLC in order to determine the amount of umbelliferone remaining in the soil. Recoveries were >90% of the umbelliferone freshly applied to the unamended or amended soils.

Data obtained in the dissipation experiment were analysed using first-order kinetic model:

$${\rm{C}}={{\rm{C}}}_{0}\cdot {{\rm{e}}}^{-k\cdot {\rm{t}}}$$

where C (mg kg^−1^) is the concentration of umbelliferone in soil at a time t (days), C_0_ is the concentration at t = 0, and *k* (day^−1^) is the first-order dissipation rate constant. Half-lives were calculated as *t*_1/2_ = 0.693/*k*.

In a separate incubation experiment, the changes in the basal respiration of the soils upon amendment with SA-HDTMA or BC were also determined. Soil respiration measurements were conducted following the alkali trapping-titrimetric procedure^51^. The method consisted of quantifying the amount of CO_2_ released by soil samples during 7 days, under conditions that were representative of those used for the umbelliferone dissipation experiment. In brief, triplicate samples of 20 g of the soils (A1 and B2), either unamended or amended with SA-HDTMA or BC at a rate of 40 mg g^−1^ and with the water content adjusted to 35% (A1) or 30% (B2), were incubated in Erlenmeyer flasks at 25 °C in the dark. After 7 days, the amount of CO_2_ released by the soil samples was determined by back-titration of vials containing 20 mL of 0.2 M NaOH that were placed inside the flasks as CO_2_ traps.

### Column leaching experiments

Leaching of umbelliferone was studied in glass columns (30 cm length × 3.1 cm internal diameter) hand-packed with soils A1 and B2, either unamended or amended with BC. Glass wool and sea sand (10 g) was placed on the bottom of the columns to avoid losses of soil and contamination of leachates with soil particles. Next, the columns were hand-packed with 20 cm of unamended or BC-amended soil. The BC-amended soil columns were prepared by mixing BC with the upper 0–5 cm of soil at a rate of 40 mg BC g^−1^ soil. Finally, sea sand (10 g) was placed on the top of the columns to minimise disturbance of the soil surface and also to allow a uniform distribution of the umbelliferone and water subsequently added to the soil columns. All leaching experiments were conducted in triplicate.

The columns were saturated by adding water to their surface (90 mL), and then were allowed to drain for 4 h. The pore volume of the soil columns, calculated by subtracting the amount of water leached from the amount added, ranged between 60 and 64 mL.

Umbelliferone was applied to the surface of the columns at a rate of 2 kg ha^−1^ as 0.15 mg of compound dissolved in 0.75 mL of methanol, which was allowed to evaporate for 1 h. If we assume a soil bulk density of 1.2 g cm^−3^ and that umbelliferone is uniformly distributed along the top 0-8 cm soil layer, an application rate of 2 kg ha^−1^ would yield the initial umbelliferone concentration used in the dissipation experiments (2 mg kg^−1^). The columns were eluted by conducting daily 3 additions of 15 mL of distilled water at 3 h intervals for a total of 12 additions in 4 days. The leachate resulting from each water addition was collected in a glass vial containing 5 mL of methanol, filtered, and analysed by HPLC to determine the concentration of umbelliferone. Both the columns and the vials used to collect the leachates were covered with aluminium foil to protect umbelliferone from light exposure. At the end of the leaching experiment, soil samples were taken from different depths of the columns (0–5, 5–10, 10–15, and 15–20 cm depth) and shaken with an 80:20 methanol:aqueous H_3_PO_4_ (pH = 2.2) mixture (100 mL) for 24 h. The suspensions were centrifuged, filtered, and analysed by HPLC to determine the amount of umbelliferone remaining at different depths of the soil columns.

### Analysis of umbelliferone

Umbelliferone was determined by HPLC using a Waters 515 HPLC Pump coupled to a Waters 2996 photodiode array detector and a Waters 717plus Autosampler injector. The analysis was carried out using a Kinetex C18 column (150 mm length × 4.6 mm internal diameter and 5 µm particle size), methanol:aqueous H_3_PO_4_ pH = 2.2 (65:35) eluent mixture at a flow rate of 1 mL min^−1^, 25 µL sample injection volume, and UV detection at 323 nm. Under these conditions, the retention time for umbelliferone was 4.7 min. The limit of quantification, calculated as the concentration resulting in a signal-to-noise ratio of 10:1, was 0.03 mg L^−1^. External calibration curves with five standard solutions between 0.1 and 6 mg L^−1^ prepared in 50:50 methanol:water were used in the calculations.

### Statistical analysis

Statistical analyses were carried out using IBM SPSS Statistics 15.0 for Windows. Standard error was used to describe variability among independent replicate samples. Differences in umbelliferone sorption, dissipation, and leaching data and in soil respiration values were compared using One-Way ANOVA followed by post-hoc Tukey’s Honestly Significant Difference test for pairwise comparison of treatments. Fitting of dissipation data to first-order kinetics was carried out using Sigma Plot for Windows Version 12.5.

## Results and Discussion

### Storage stability of umbelliferone

Since allelochemicals can display a poor stability in aqueous solution and/or soil extracts, it is important to test their storage stability to avoid analytical artefacts when conducting experiments to assess their behaviour in soils^24,49^. The stability of umbelliferone in different water/methanol mixtures exposed to light and under dark conditions is illustrated in Supplementary Fig. S1a. Under light exposure, the concentration of umbelliferone progressively decreased with time when the amount of methanol in the mixture was ≤10%; however, it remained stable when the amount of methanol was increased to 50% and also in all water/methanol mixtures when these were protected from light.

The stability of umbelliferone in a soil extract containing different amounts of methanol was also studied (Supplementary Fig. S1b). Under light exposure, the concentration of umbelliferone rapidly decreased in all cases, although increasing the amount of methanol reduced the dissipation of the compound in the soil extract. Protection from light further increased the stability of umbelliferone in the soil extracts; more than 90% of the umbelliferone initially present remained in solution after 8 days when the methanol content was 10 or 50%.

On the basis of the results of the stability experiments, we concluded that umbelliferone aqueous solutions were unstable when exposed to light and to water-extractable soil constituents, presumably because of its susceptibility to photochemical and microbial degradation processes, respectively. Nevertheless, the addition of methanol at a rate ≥ 10% combined with storage in the dark stabilised the compound for at least 8 days (Supplementary Fig. S1b). Consequently, all solutions of umbelliferone to be analysed during the sorption and leaching experiments conducted in this work were stabilised with an amount of methanol higher than 10% and kept in the dark to prevent any disappearance of compound during storage (<1 week) prior to analysis.

### Umbelliferone sorption on soils and sorbents

**Figure 2:**
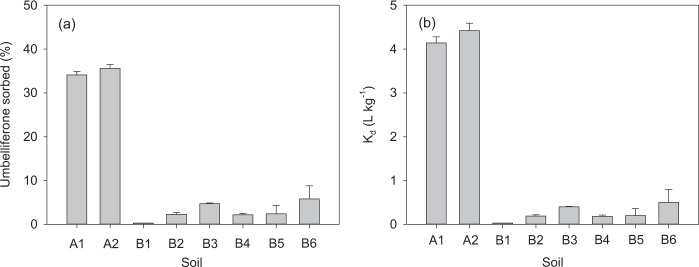
(**a**) Percentage of umbelliferone sorbed on soils and (**b**) corresponding sorption distribution coefficients, K_d_. Measurements were conducted at an initial umbelliferone concentration of 2 mg L^−1^ and with a soil to solution ratio of 1 g:8 mL. Error bars indicate ± s.e.m. (n = 3).

Umbelliferone sorption on soils was evaluated using eight pre-sterilised soil samples that covered a range of physicochemical characteristics representative of the Mediterranean area (Supplementary Table S1). Sorption was noticeable only for the acid soils tested (A1 and A2), while all alkaline soils (B1-B6) displayed very low sorption values (%Ads < 5%, *K*_d_ < 0.5 L kg^−1^) (Fig. 2). The amount of umbelliferone desorbed from soils A1 and A2, by addition of methanol immediately after the 24 h sorption experiment, accounted for more than 90% of the compound depleted from solution during the sorption step. This confirmed that sorption measurement was accurate and that chemical or biological transformation processes did not interfere during the sorption experiment. The phenolic group of umbelliferone (Fig. 1) has a p*K*_a_ ≈ 7.5, and so should have remained mainly undissociated at the pH of the acid soils (pH = 5.5–6.0), favouring sorption. Conversely, it should have become partially ionised at the pH of the alkaline soils (pH = 8.0–8.6), and at such pH levels umbelliferone anions were probably repelled by the negatively charged surfaces of soil clays and organic matter, thus explaining low sorption values^52,53^.

**Figure 3:**
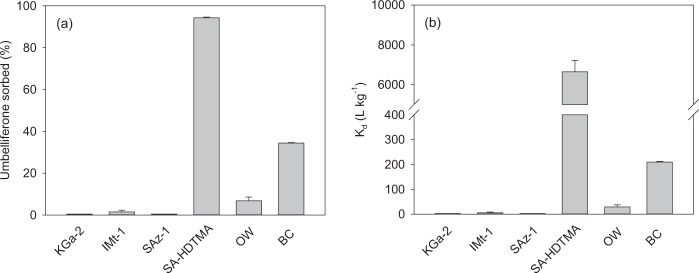
(**a**) Percentage of umbelliferone sorbed on different sorbents and (**b**) corresponding sorption distribution coefficients, K_d_. KGa-2: Georgia kaolinite; IMt-1: Montana illite; SAz-1: Arizona montmorillonite; SA-HDTMA: hexadecyltrimethylammonium-modified Arizona montmorillonite; OW: olive-mill waste; BC: biochar from OW. Measurements were conducted at an initial umbelliferone concentration of 2 mg L^−1^ and with a sorbent to solution ratio of 20 mg:8 mL. Error bars indicate ± s.e.m. (n = 3).

The results of umbelliferone sorption on different sorbent materials are summarised in Fig. 3. All sorbents, except KGa-2 (pH = 6.6), yielded alkaline pH values ranging from 7.8 to 9.5 (Supplementary Table S2). The affinity of umbelliferone for the sorbents decreased in the following order: SA-HDTMA $\gg$ BC > OW > SAz-1 ≈ IMt-1 ≈ KGa-2. Thus, the affinity of umbelliferone for the organoclay SA-HDTMA (%Ads = 94 ± 1%) was greater than for the unmodified clay (SAz-1). Similarly, the pyrolysed OW (BC) displayed increased sorption (%Ads = 34 ± 1%) compared to its feedstock (OW) material (%Ads = 7 ± 2%). The sorption percentages for SAz-1, KGa-2 and IMt-1 were very low (<5%). As for the soils, the amount of umbelliferone desorbed from SA-HDTMA and BC upon the addition of methanol immediately after the 24 h sorption experiment accounted for most (>85%) of the compound depleted from solution during the sorption step. The nanoengineered sorbents, SA-HDTMA and BC, were thus those displaying the greatest affinity for umbelliferone, with the organoclay (SA-HDTMA) showing significantly (*p* < 0.001) greater performance than the biochar (BC) sample at the experimental conditions used.

### Umbelliferone sorption mechanisms on SA-HDTMA and BC

The incorporation of HDTMA cations in the interlayer of SAz-1 is known to increase the organic carbon content and the basal spacing value of the clay, creating a paraffin-like interlayer structure with hydrophobic properties and high affinity for organic compounds^37^. For the SA-HDTMA sample used in this work, the organic carbon content increased from < 0.1% for the untreated clay to 22.1 ± 0.1% for the modified clay, and the clay basal spacing was expanded from 15.2 Å to 23.0 Å (Supplementary Fig. S2). These changes increased the affinity of the clay for umbelliferone (Fig. 3), probably by favouring hydrophobic interactions between the alkyl chains of the organic modifier and the aromatic moieties of the allelochemical (Fig. 1). Furthermore, the HDTMA cations also provide the clay mineral surface with positively charged quaternary ammonium groups (Supplementary Fig. S2) and some of these are known to constitute potential sites for the sorption of organic anions^46,47^. Given that umbelliferone should have largely existed as anionic species at the pH of the equilibrated suspension of SA-HDTMA (pH = 7.9, Supplementary Table S2), electrostatic (ionic) attraction between positively charged quaternary ammonium groups of SA-HDTMA and umbelliferone anions could have contributed to the sorption mechanism.

Supplementary Fig. S3 shows the FT-IR spectrum of SA-HDTMA treated with a concentrated umbelliferone solution along with the spectra of the untreated organoclay and pure umbelliferone. The spectra showed two noticeable effects. First, the absence of the O-H stretching vibration band of umbelliferone at 3140 cm^−1^ in the spectrum of the SA-HDTMA-umbelliferone complex suggested that umbelliferone could have predominated in the sorbed state as ionised species. Second, when comparing the spectra of pure and sorbed umbelliferone, small changes in the position of the C=C stretching vibrations at 1400–1600 cm^−1^ were observed, suggesting involvement of the aromatic rings of the allelochemical in the interaction with the organoclay. Ionic attraction and aromatic ring-alkyl chain hydrophobic interactions have previously been suggested as cooperative mechanisms driving the sorption of aromatic acids by paraffinic organoclays^46,53^.

In the case of the BC sample, it is known that biochars produced at low pyrolysis temperatures (e.g., 400 °C) contain, along with newly-formed condensed carbon and porosity, surface polar functional groups from the feedstock (Supplementary Fig. S4), which allow both hydrophobic and polar interactions with ionisable organic compounds^33^. Since pyrolysis of biomass leads to a reduction in abundance of surface polar functional groups, the increase in aromaticity and porosity probably explained the high sorption of umbelliferone by the BC sample^54^. Lian and Xing^33^ and Kah *et al*.^55^ indicated that electrostatic repulsions, charge assisted H-bonds, cation bridging, and anion-π bonds dominate the interaction of organic acids with carbonaceous sorbents at high pH values, such as that measured in this study for the BC-umbelliferone suspension (pH = 9.5, Supplementary Table S2). Unfortunately, the FT-IR spectra were inconclusive about the relative importance of the above-mentioned interaction mechanisms, because the amount of umbelliferone sorbed in the complex prepared for the FT-IR spectroscopy study was too low (11 mg g^−1^) to produce significant changes in the spectrum of BC (Supplementary Fig. S3).

### Effect of SA-HDTMA and BC addition on umbelliferone sorption by selected soils

On the basis of the results presented in the preceding sections, the nanoengineered sorbents, SA-HDTMA and BC, were selected as soil amendments to evaluate their effect on umbelliferone sorption and persistence in two distinct soils, one acid (A1) and one alkaline (B2). The results of the sorption experiments are given in Table 1.

**Table 1:** Umbelliferone sorption percentages (%Ads), pH values, and experimental (*K*_d_) and calculated (*K*_d-calc_) distribution coefficients for unamended and SA-HDTMA- and BC-amended soil samples ( ± s.e.m., n = 3)

Soil	%Ads	pH	*K*_d_ (L kg^−1^)	*K*_d-calc_ (L kg^−1^)
A1 (unamended)	36.6 ± 2.1a	5.7	4.6 ± 0.4ad	—
A1 + BC	61.1 ± 2.4b	6.9	12.2 ± 1.2b	12.5
A1 + SA-HDTMA	71.1 ± 1.1c	5.6	19.0 ± 1.0c	260.2
B2 (unamended)	3.3 ± 2.1d	8.1	0.3 ± 0.2e	—
B2 + BC	17.0 ± 0.6 fe	8.0	1.6 ± 0.1e	8.3
B2 + SA-HDTMA	20.9 ± 1.6f	8.1	2.0 ± 0.2de	256.0

Different letters in the same column indicate that differences between treatments are statistically significant (*p* < 0.05). *K*_d-calc_ = *K*_d-soil_ *f*_soil_ + *K*_d-amend_ *f*_amend_, where *K*_d-soil_ and *K*_d-amend_ are the individual *K*_d_ values measured for umbelliferone sorption on soil and amendment, and *f*_soil_ and *f*_amend_ are the fraction of soil and amendment in the mixture, respectively^59,60^.

The addition of BC and SA-HDTMA at a rate of 4% led to an important increase in the sorption of umbelliferone by both soils. The *K*_d_ value for umbelliferone in unamended soil A1 (4.6 L kg^−1^) increased to 12.2 L kg^−1^ upon amendment with BC and to 19.0 L kg^−1^ upon amendment with SA-HDTMA. For soil B2, the low sorption of umbelliferone in the unamended soil (*K*_d_ = 0.3 L kg^−1^) increased upon the addition of BC and SA-HDTMA, to *K*_d_ values of 1.6 and 2.0 L kg^−1^, respectively. The fact that the SA-HDTMA-amended soils showed higher sorption than the BC-amended soils (Table 1) reflected the sorptive properties of the pure amendments (Fig. 3). Nevertheless, when we compared the *K*_d_ values obtained for the amended soils with the values predicted assuming linear and independent sorption behaviour for the soil and the amendment in the mixtures (*K*_d-calc_), we concluded that the increase in sorption produced by the amendments was, in general, less than expected from the individual sorption constants (Table 1). This result indicated that the amendments could have lost some of their sorption performance in the presence of the soils. Most likely, the interaction of the amendments with soil constituents resulted in competition with umbelliferone for sorption sites and/or blockage of access to such sorption sites, partially reducing the performance of the amendments as sorbents of umbelliferone^25,47^. Data in Table 1 show that this effect was soil- and amendment-dependent, and more pronounced for SA-HDTMA and for the alkaline soil (B2).

### Dissipation of umbelliferone in unamended and amended soils

**Figure 4:**
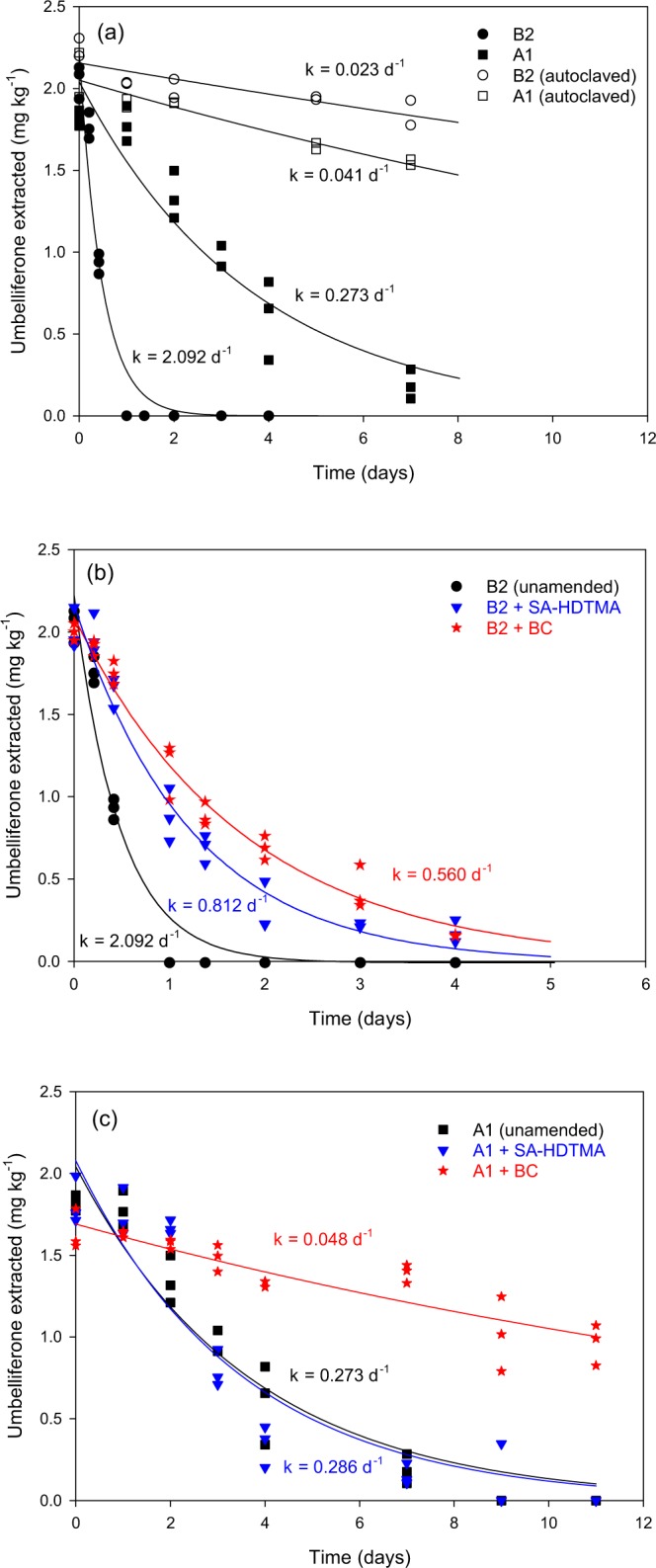
Umbelliferone dissipation curves in sterilised and nonsterilised soils (**a**) and effect of the addition of SA-HDTMA and BC at a rate of 4% on the dissipation of umbelliferone in non-sterilised soils B2 (**b**) and A1 (**c**). Symbols are experimental data, whereas lines represent the fittings to single first-order dissipation.

After 7 days of incubation, the residual percentage of umbelliferone in nonsterilised A1 and B2 soil had decreased to 10 and 0%, respectively, while in the sterilised controls the residual percentages of umbelliferone remained >75% (Fig. 4a). Thus, the degradation of umbelliferone in the soils was fast and mainly microbial-mediated, in agreement with data recently reported by Guo *et al*.^22^. The values of the first-order dissipation rate constants showed that dissipation was faster in B2 soil (*k* = 2.092 day^−1^) than in A1 soil (*k* = 0.273 day^−1^), with half-lives (*t*_1/2_) of 0.3 and 2.5 days, respectively. Along with an intrinsic reduced biodegradative activity of the acid soil (A1), supported by a low basal respiration (Supplementary Table S3), sorption (Fig. 2) could have contributed to delay the degradation of umbelliferone in this soil, as sorption processes are known to reduce the bioavailability of organic compounds and protect them from microbial degradation^56^.

The persistence of umbelliferone in soil B2 increased by the addition of both SA-HDTMA (*k* = 0.812 day^−1^) and BC (*k* = 0.560 day^−1^). At t = 1 day, umbelliferone had completely dissipated from unamended B2 soil, whereas more than 40% of the amount initially added still remained in SA-HDTMA- and BC-amended soil (Fig. 4b). From t = 0.4 to 4 days, umbelliferone residues in the soil amended with SA-HDTMA and BC were significantly greater (*p* < 0.05) than those in unamended soil. Since the effect of SA-HDTMA and BC on the respiration of the soils used in the present study did not suggest an inhibitory effect of the amendments on the soil microbial activity (Supplementary Table S3), the increase in sorption of umbelliferone produced by the amendments (Table 1) probably reduced its availability to microorganisms and increased its persistence in the soil. According to the experimental *K*_d_ values reported in Table 1, we estimated that at the beginning of the dissipation experiment the amount of umbelliferone sorbed to the solid phase accounted for 50% for the unamended soil B2, and increased to 85 and 87% for BC-amended and SA-HDTMA-amended soil B2, respectively. The limited amount of umbelliferone available in solution in the amended soil probably reduced its availability to microorganisms and increased its persistence in the soil. For soil A1, we observed a significant increase in the persistence of umbelliferone (*p* < 0.05 for residues between t = 3 and 11 days) only upon amendment with BC (Fig. 4c). According to the (high) *K*_d_ values reported in Table 1, the percentage of umbelliferone sorbed were estimated to be 93% for unamended soil, 97% for BC-amended soil, and 98% for SA-HDTMA-amended soil. The high fraction of sorbed umbelliferone in the unamended soil probably made the effect of the amendments on the bioavailability of the compound less noticeable than in soil B2.

It is interesting to note that, conversely to what predicted by the *K*_d_ values reported in Table 1, the increase in persistence of umbelliferone in both soils produced by the addition of BC was greater than that produced by the addition of SA-HDTMA. In this regard, Gámiz *et al*.^47^ monitored the changes in sorption of the phytohormone abscisic acid with time in a soil amended with organoclay and a high pyrolysis temperature apple wood biochar and their results indicated some loss of the sorption capacity with time for the organoclay-amended soil and, conversely, increasing sorption of abscisic acid with time in BC-amended soil. These distinct time-dependent sorption behaviours observed by Gámiz *et al*.^47^ suggested external surface sorption mechanism for the organoclay and slow kinetics (potentially pore diffusion) in BC-amended soil^57,58^. This would also explain the higher potential of BC for protecting umbelliferone from biodegradation in the soils tested in this study, which was particularly noticeable at longer incubation times.

### Effect of BC addition on umbelliferone leaching in soils

On the basis of its greater ability to increase the persistence of umbelliferone in the soils used in the incubation experiments, BC was selected as amendment to evaluate its effect on the mobility of umbelliferone in column leaching experiments with the same soils. In agreement with the low *K*_d_ value measured for soil B2 in the sorption study (Table 1, Fig. 2), umbelliferone was detected only in leachates from unamended B2 soil columns (Table 2). The total amount of umbelliferone leached was low (14%), probably because umbelliferone rapidly degraded during leaching through this soil (Fig. 4a). Umbelliferone was not detected in the leachates from the columns packed with unamended soil A1 or with BC-amended soils (Table 2), for which the compound presented high *K*_d_ values (Table 1).

**Table 2:** Umbelliferone leached, extracted from the soil, and not recovered after the leaching experiment conducted in unamended and BC-amended soil columns (±s.e.m., n = 3).

Soil	Leached (%)	Extracted (%)	Not recovered (%)
A1 (unamended)	0	16 ± 4	84 ± 4
A1 + BC	0	57 ± 9	43 ± 9
B2 (unamended)	14 ± 2	6 ± 1	80 ± 2
B2 + BC	0	23 ± 9	77 ± 9

**Figure 5:**
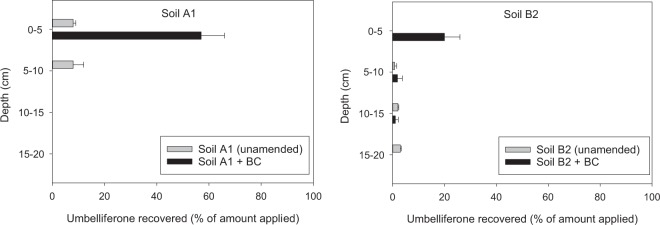
Umbelliferone extracted from different depths of unamended and BC-amended soil columns at the end of the leaching experiment. Error bars indicate ± s.e.m. (n = 3).

At the end of the leaching experiment (240 mm of water added within 4 days), the soil in each column was analysed to determine umbelliferone residues at different depths. For both soils, the presence of BC led to an increase in the amount of umbelliferone remaining in the soils and accumulation of the compound in the top 0–5 cm (BC-amended) soil layer (Table 2, Fig. 5). This effect was more pronounced for the acid soil (A1), for which 57% of the applied umbelliferone was recovered from the 0–5 cm layer (Fig. 5). Clearly, sorption of umbelliferone in the BC-amended (0–5 cm) soil layer reduced its leaching and delayed its degradation compared to the unamended soils.

## Conclusions

The results of this work show, in summary, that nanoengineered sorbents can be useful as soil additives to modulate the mobility and persistence of allelochemicals in soils, which may be applied to overcome some of the limitations associated with the use of allelochemicals as environmentally friendly crop protection substances. Specifically, we showed how organoclays and biochars can be designed at the nanoscale to increase the sorption and reduce the mobility and degradation of the allelochemical umbelliferone in two distinct soils. The observed effects were both sorbent- and soil-dependent, and particularly pronounced for biochar added to acid soil. Changes in the sorption properties of the sorbents in the presence of soil and sorption evolution with time were identified as two important factors determining their effect on the mobility and persistence of umbelliferone. Controlling these two factors seems important to optimise the performance of organoclays and biochars after their addition to soils, and presumably, to achieve the desired effect on the biological activity of allelochemicals, in the context of taking advantage of their bioactivity for crop protection.

## Supplementary information

Supplementary information

## Data Availability

The datasets generated during the current study are available from the corresponding author on reasonable request.
