# Supplementary material for: Sorption, persistence, and leaching of the allelochemical umbelliferone in soils treated with nanoengineered sorbents
Source: Sci Rep. 2019 Jul 5;9:9764. doi: 10.1038/s41598-019-46031-z (PMC6611869; doi:10.1038/s41598-019-46031-z)
Supplement: Supplementary file 1 — Supplementary information [file 41598_2019_46031_MOESM1_ESM.docx]

Supplementary information for

**Sorption, persistence, and leaching of the allelochemical umbelliferone in soils treated with nanoengineered sorbents**

Miguel Real, Beatriz Gámiz, Rocío López-Cabeza and Rafael Celis*

*Instituto de Recursos Naturales y Agrobiología de Sevilla (IRNAS), CSIC, Avenida Reina Mercedes 10, 41012 Sevilla, Spain*

**Table S1.** Main properties of the soils used.

| **Soil** | **Texture** | **Sand** | **Silt** | **Clay** | **Clay mineralogy^a^** | | | **CaCO_3_** | **OC^b^** | **pH^c^** |
| --- | --- | --- | --- | --- | --- | --- | --- | --- | --- | --- |
|  |  | **(%)** | **(%)** | **(%)** | **K (%)** | **I (%)** | **M (%)** | **(%)** | **(%)** |  |
| **A1** | **sandy loam** | **68** | **24** | **8** | **50** | **50** | **0** | **< 0.5** | **1.23** | **6.0** |
| A2 | loam | 51 | 40 | 9 | n.a.^d^ | n.a. | n.a. | 0.8 | 0.66 | 5.5 |
| B1 | sand | 89 | 5 | 5 | 20 | 44 | 36 | 5.9 | 0.23 | 8.3 |
| **B2** | **sandy loam** | **74** | **7** | **19** | **29** | **18** | **53** | **4.8** | **0.72** | **8.0** |
| B3 | sandy loam | 63 | 19 | 18 | 5 | 7 | 88 | 17.9 | 1.18 | 8.2 |
| B4 | loam | 50 | 29 | 21 | 29 | 19 | 52 | 32.5 | 1.17 | 8.3 |
| B5 | loam | 48 | 28 | 23 | 20 | 80 | 0 | 3.3 | 0.79 | 8.6 |
| B6 | clay loam | 22 | 51 | 27 | 4 | 33 | 63 | 23.8 | 1.37 | 8.3 |

^a^ relative percentages of kaolinite (K), illite/mica (I) and montmorillonite (M) in the soil clay fractions.

^b^ organic carbon.

^c^ measured in a 1 g:2.5 mL soil:water suspension.

^d^ not available

**Table S2.** pH values of the equilibrated suspensions in the umbelliferone sorption measurements with sorbent materials.

|  | **KGa-2** | **IMt-1** | **SAz-1** | **SA-HDTMA** | **OW** | **BC** |
| --- | --- | --- | --- | --- | --- | --- |
| **pH** | 6.6 | 9.0 | 7.8 | 7.9 | 9.5 | 9.5 |

**Table S3.** Soil respiration values for the unamended and SA-HDTMA- and BC-amended soils A1 and B2 (± s.e.m., n = 3). Different letters in the same column indicate that differences between treatments are statistically significant (p < 0.05).

| **Treatment** | **Soil respiration (mg CO_2_ kg^-1^ soil per week)** | |
| --- | --- | --- |
|  | **Soil A1** | **Soil B2** |
| Unamended soil | 554 ± 88a | 928 ± 15a |
| Soil + SA-HDTMA | 557 ± 60a | 854 ± 68a |
| Soil + BC | 2075 ± 19b | 1525 ± 48b |

**Figure S1.** Stability of umbelliferone solutions exposed to light and in the dark: (a) umbelliferone in water/methanol mixtures, (b) umbelliferone in aqueous soil extract/methanol mixtures. The soil extract was the (filtered) solution resulting from shaking 50 g of soil B2 with 100 mL of water for 24 h. The initial concentration of umbelliferone was 2 mg L^-1^.

**Figure S2.** Some characteristics of the organoclay SA-HDTMA used in this work: (a) X-ray diffractogram, compared to that of the unmodified clay (SAz-1), showing the basal diffraction at 23.0 Å and schematic structure of the interlayer arrangement of the HDTMA cations, (b) total carbon content compared to that of the unmodified clay (SAz-1), and (c) FT-IR spectra of SA-HDTMA and SAz-1 showing the absorption bands of the alkylammonium cations in SA-HDTMA.

**Figure S3.** FT-IR spectra of: (a) pure umbelliferone, (b) SA-HDTMA, (c) SA-HDTMA-umbelliferone complex (53 mg umbelliferone g^-1^ organoclay), (d) BC, and (e) BC-umbelliferone complex (11 mg umbelliferone g^-1^ biochar).

**Figure S4.** Some characteristics of the biochar (BC) sample used in this work: a) SEM micrograph showing details of the morphology of a BC particle, (b) total carbon content compared to that of its feedstock material (OW), and (c) FT-IR spectra of BC and OW showing absorption bands of main functionalities.
